# Turning Agricultural Waste into Useful Biochemicals and Biofuels Through Biochemical Engineering and Biotechnological Processing

**Published:** 2025-06

**Authors:** Jun Wei Lim

**Affiliations:** 1 HICoE-Centre for Biofuel and Biochemical Research, Institute of Sustainable Energy and Resources, Department of Applied Science, Universiti Teknologi PETRONAS, 32610, Seri Iskandar, Perak Darul Ridzuan, Malaysia; 2 Department of Biotechnology, Saveetha School of Engineering, Saveetha Institute of Medical and Technical Sciences, Saveetha University, 602105, Chennai, India

For decades, agriculture has been the main support of the economies of many countries, especially in Southeast Asia such as Thailand, Vietnam, Indonesia, Cambodia and Malaysia, which depend heavily on numerous farming activities. Accordingly, agricultural land accounts for about 20 to 50 % of the total area of these countries. Recently, the intensive growth of agricultural production has gradually led to the generation of large amounts of waste. This waste includes crop residues, especially by-products and nutrient-rich agricultural wastewater. When this waste is poorly managed, it leads to severe environmental pollution, including greenhouse gas emissions, contamination of surrounding water bodies, and ultimately the ecosystem disruption that will eventually affect human well-being. For example, the anaerobic degradation of agricultural residues releases methane, a potent greenhouse gas. Moreover, the untreated wastewater from processing, such as palm oil mill effluent from palm mills, is usually laden with excessive organic loads and nutrients that cause serious water pollution and eutrophication in receiving water bodies, posing a threat to aquatic life.

Despite these challenges in dealing with agricultural waste, some prospects and opportunities arise from sustainable management. The organic waste generated on farms and in the processing industry can be converted into valuable biochemicals and biofuels *via* advanced and innovative biochemical engineering and biotechnological processes. Accordingly, this special issue explores cutting-edge techniques that will not only reduce waste-related pollution, but also contribute to a circular bioeconomy. By applying sustainable processes related to microbial fermentation, enzymatic conversion, genetic modification and biorefinery concepts, researchers reveal the potential of agricultural waste as renewable feedstock for real industrial applications. These approaches align directly with the Principles of Green Chemistry (*1*) and United Nations Sustainable Development Goals (*2*), particularly those focusing on responsible consumption and production, climate action, and affordable and clean energy. Among the key strategies, enzymatic conversion has been widely adopted because of its ability to efficiently break down the complex lignocellulosic biomass waste into fermentable sugars. The improvements in enzymatic bioprocesses have further increased the cost-effectiveness in the production of fermentable sugars, making it a feasible pathway for the production of biochemicals and biofuels. In addition, fermentation bioprocesses are also being expanded to convert agricultural waste into high-value biochemicals such as lactic acid, succinic acid, bioethanol, *etc*. Another promising prospect is the use of microbial fuel cells in which the metabolic activities of bacterial cells are utilised to simultaneously generate electricity and treat organic wastewater. This dual-function bioprocessing is particularly applicable in industries that produce liquid organic waste, namely, palm oil mills and food processing factories, whose effluents pose significant disposal challenges due to high load of chemical oxygen demand and biological oxygen demand. In supplementing microbial fuel cell technology, the bioaugmentation approach can further promote the degradation of organic waste by inoculating and mixing with specialised microbial consortia to accelerate the decomposition and anaerobic bioprocesses. On another note, the effectiveness of composting and vermicomposting in the treatment of agricultural waste is another important point for reducing biomass residues through a biological process that is naturally sustainable. Genetic modification and metagenomics can further expand the scope of agricultural waste treatment. By recognising and modifying cellular microbial pathways, microorganisms such as bacteria can be tailored to break down recalcitrant structures in biomass waste more efficiently or synthesise targeted biochemicals. Meanwhile, microalgal biotechnology also offers a sustainable method for recovering nutrients from agricultural wastewater, with the microalgal cells serving as biofactories for the production of lipids, proteins and other biocompounds. The microalgae are also able to utilize carbon dioxide during photosynthesis when they are exposed to sunlight, reducing the carbon dioxide emission of industries that grow microalgae and contribute to decarbonisation with a smaller carbon footprint. The biological nitrogen fixation and biofiltration systems are also being explored for low-energy solutions, thereby reducing the overall environmental footprint of agricultural emissions. Recently, the growing field of bioplastic production has been revitalised. In this case, polymers derived from agricultural waste provide a renewable alternative to replace petroleum-based plastics. This environmentally friendly alternative is important to resolve the long-standing threat posed by plastics, especially pollution with microplastics and nanoplastics. Finally, the concept of biorefineries should be strongly advocated as a holistic framework for the integration of multiple waste-to-value bioprocesses and biotechnologies to ensure negligible resource loss while maximising socioeconomic return.

Indeed, the global community should constantly strive for decarbonisation to sustain the well-being of future generations. The transition from the disposal of agricultural waste to the recovery of various resources is not merely an environmental obligation but also proffers an economic opportunity for societies by fostering resilience in advanced agricultural systems while reducing traditional reliance on fossil fuels. Accordingly, the innovations compiled in this special issue can provide actionable insights for global policymakers, industrial leaders and researchers pursuing a similar interest in green agricultural farming. I would also like to extend my deepest gratitude to all the authors, reviewers and the editorial team whose combined efforts have made this special issue an important contribution to the fields of biochemical engineering and biotechnological processing. Continuous interdisciplinary research and management of agricultural waste by biotechnological methods have brought the idea of sustainable and waste-free agriculture within reach. This special issue will now serve as a catalyst for upcoming innovations in utilising agricultural waste, *i.e*. a novel step towards greener and more sustainable agriculture.

## References

[1] 12 Principles of Green Chemistry [Internet]. ACS Chemistry for Life; 2025. Available from: https://www.acs.org/green-chemistry-sustainability/principles/12-principles-of-green-chemistry.html.

[2] Sustainable Development Goals. 17 Goals to Transform Our World [Internet]. United Nations; 2025. Available from: https://www.un.org/sustainabledevelopment/.

